# Distribution pattern of poliovirus potentially infectious materials in the phase 1b medical laboratories containment in conformity with the global action plan III

**DOI:** 10.1186/s12889-018-6183-1

**Published:** 2018-12-13

**Authors:** Bassey Enya Bassey, Fiona Braka, Faisal Shuaib, Richard Banda, Sisay Gashu Tegegne, Johnson Muluh Ticha, Walla Hamisu Abdullalhi, Olatunji Mathew Kolawole, Yusuf Kabir

**Affiliations:** 1 World Health organization (WHO) Nigeria Country office, UN House, plot 617/618, Diplomatic Drive, Central Business District, PMB 2861, Garki, Abuja, Nigeria; 2 National Primary Health care Development Agency, Abuja, Nigeria; 3 Department of Microbiology, Faculty of Life Sciences, University of Ilorin, Ilorin, Kwara State Nigeria

**Keywords:** GAP III, AFP surveillance, Poliovirus infectious materials, Polio eradication

## Abstract

**Background:**

The containment of poliovirus infectious/potentially infectious materials in all biomedical facilities in Nigeria remain crucial to maintaining gains recorded towards polio eradication. Activities involved in the Nigerian Poliovirus type 2-laboratory containment survey in line with the 3rd Global Action Plan III (GAP III) for poliovirus containment are documented in this study. Through these activities, the overall preparedness for poliovirus eradication in Nigeria is assessed.

**Method:**

A cross-sectional survey was conducted from 19th September-31st October 2016 using structured Laboratory survey and inventory (LSI) questionnaires uploaded onto the SPSS software package in 560 biomedical facilities classified either as high risk or medium risk facilities across the 6 zones in Nigeria.

**Results:**

In total, 560 biomedical facilities were surveyed in Nigeria in conformity with the GAP III. In total, 86% of the facilities surveyed were with laboratories while 14% were without laboratories.

Twelve laboratories with poliovirus potentially infectious materials were identified in this exercise. In total, 50% of the 12 laboratories were under the ministry of education for research purposes. While 33% were among those laboratories surveyed in the phase 1a exercise without any recorded inventory, but have acquired some since the phase 1a survey.

A total of 13,484 poliovirus infectious materials were found in the 12 laboratories. Only 8% of the materials were immediately destroyed while the remaining materials (62%) were found in Oyo and Borno states scheduled for destruction within 3–4 months according to WHO protocol for destruction of poliovirus infectious materials.

**Conclusion:**

This study has revealed the successful containment of all poliovirus infectious materials in the laboratories surveyed. It has also revealed some surveillance gaps. We recommend that the surveillance system be improved to maintain the gains from the containment exercise and avoid reintroduction of infectious materials into biomedical facilities. This reduces the chances of viral reintroduction to the population in general.

## Background

The world will be declared poliovirus free when the Global Certification Commission (GCC) is satisfied that all World Health Organization (WHO) regions have documented the absence of poliovirus for at least three years [1, 2].

In the past 11 years, Nigeria has made remarkable progress towards polio eradication with the striking decline in the number of Wild Polioviruses (WPV) from 1122 cases in 2006 to just three cases in 2016 [3–5]. The number of circulating Vaccine-Derived Poliovirus (cVDPV) cases has also declined from 27 cases in 2010 to just one case in 2016 [3, 6]. There has also been a notable improvement in the sensitivity of the Acute Flaccid Paralysis (AFP) surveillance system in the past year. Specifically, a total of 13,029 AFP cases and a 21.7 Non-polio (NP)- AFP rate was recorded between January and September 2016 in comparison to the 10,870 AFP cases and 18.7 NP-AFP rate recorded in 2015 [7]. Nigeria also participated in the globally synchronized switch from trivalent Oral Polio Vaccine (tOPV) to bivalent Oral Polio Vaccine (bOPV) in April 2016 after WPV2 was declared eradicated in 2015 by the GCC. [8, 9]

The safe handling and containment of unwanted poliovirus type 2 and indeed all poliovirus infectious/ potentially infectious materials in all laboratories/biomedical facilities and health facilities in the country is therefore crucial if gains made towards polio eradication are to be maintained because it minimizes the risk of facility-associated poliovirus reintroduction [10, 11].

The Global Action Plan III (GAP III) for poliovirus containment champions the process of minimizing poliovirus facility-associated risks [12, 13]. All WHO member states implemented appropriate containment of WPV type 2 in essential laboratories and vaccine production facilities by the end of 2015 in preparation for the tOPV-bOPV switch. Biomedical facilities/laboratories and Health facilities (HFs) were then surveyed and checked in 2016 for compliance with the destruction of all tOPV/monovalent Oral Polio Vaccine type 2 (mOPV2) and containment/destruction of all poliovirus infectious materials months after the global withdrawal of Oral Polio Vaccine type2 (OPV2) in line with the GAP III.

The GAP III is divided into three phases in recognition of the changing nature of the risks during different stages of the eradication programme. Implementation of the GAP III’s phase I in Nigeria was down in two strata: Phase 1a, which place in 2015 and phase 1b in 2016. Nigeria implemented part of the phase 1b when she updated the national list of laboratories and facilities after the phase 1a exercise, surveyed some selected biomedical laboratories (high risk, veterinary and research institutions, medium and low risk laboratories, those facilities with inventory from the phase 1a, new facilities and those that initially decline survey in phase 1a) and identified those with poliovirus or poliovirus potentially infectious materials for prompt destruction.

Here, we document the activities involved in the Nigerian Poliovirus type 2-laboratory containment survey in line with the GAP III. Through the activities involved in this exercise, we also access the overall preparedness for poliovirus eradication in Nigeria as she progresses towards a polio free certification.

## Method

### Study design

We conducted a cross-sectional survey was conducted from 19th September-31st October 2016 (as part of the phase 1b of the GAP III on laboratory containment of the poliovirus) using structured Laboratory survey and inventory (LSI) questionnaires uploaded onto the SPSS software package in 560 biomedical facilities classified either as high risk or medium risk facilities across the six geopolitical zones in Nigeria. Those not captured in the phase 1a and those initially retaining poliovirus or poliovirus potentially infectious materials in phase 1a were also surveyed.

The laboratory/biomedical facility survey was conducted in such a way that most activities occurred at the biomedical facilities/ laboratory. Coordination of activities therein was done at different levels but final coordination was done at the National level. It involved different activities that were monitored using a standardized questionnaire. Key activities conducted were: establishment of laboratory/biomedical facility list, survey of such facilities classified as high risk and those initially retaining poliovirus or poliovirus potentially infectious materials, destruction of unwanted poliovirus type 2 infectious/potentially infectious materials and validation which was done by WHO staff, six Zonal consultants (ZCs) (representing the six geopolitical zones in the country), the National Coordinator (NC) and members of the National Task Force (NTF).

### Generation of laboratory/biomedical facility list

The National laboratory list generated in the phase 1a exercise was the major source from which selected laboratories were picked for the phase 1b containment activity. The list included facilities with any capacity to handle biological materials, including diagnostics, research, teaching and industrial production.

Biomedical facilities to be surveyed in phase 1b were selected based on those classified in phase 1a activity as outlined: high risk laboratories, those initially with inventory of infectious materials, veterinary institutions, teaching Hospitals/Federal Medical Centres, research institutions, medium risk laboratories, low risk laboratories, new facilities to be found in the field and cold stores. Information on new facilities was obtained from professional group meetings, Disease Surveillance and Notification Officers (DSNOs), National and State Chapters of the Association of Medical Laboratory Scientists in Nigeria (AMLSN) and Guild of Medical Laboratory Directors in Nigeria.

The NC was responsible for maintaining and updating the generated list as the containment activities progressed.

### Data collection

The laboratory scientist completed the LSI survey questionnaires at the laboratory/biomedical facility. Immediate retrieval and validation of the completed survey form was done. Endorsed facility visitation/evidence of destruction forms were deposited in the laboratory/biomedical facility to provide evidence that such facility have been surveyed by the ZCs and WHO staff.

Laboratories/biomedical facilities with poliovirus or poliovirus potentially infectious materialscompleted the inventory form of the LSI and were immediately validated by the ZCs and WHO staff.Delisting of endorsed laboratory/biomedical facility followed this. Note that validation of laboratories surveyed for poliovirus potentially infectious materials in the country was done solely by the six ZCs covering the six geopolitical zones under the supervision of the NC.

### Completeness of response

Almost all the laboratories/biomedical facilities initially surveyed responded to the questionnaires. Follow-up attempts using telephone calls, text messages and physically going back to the laboratories to remind them of completion of the questionnaires were carried out by the ZCs and WHO staff.

### Quality of survey

The completed LSI forms were retrieved and crosschecked by the ZCs and WHO staffs to make sure all variables were completed and validation was carried out. The retrieved validated forms were submitted weekly in WHO state offices of the survey.

The completed survey forms were double-checked by WHO state coordinators for correctness before filing in the state offices. The NC analysed data from the survey weekly.

Also, laboratories that initially declined during the phase 1a and 1b activities were surveyed again while survey forms yet to be retrieved from laboratories were collected and submitted timely and accordingly.

The NC also randomly selected laboratories/biomedical facilities in all six zones and physically validated the information provided in the completed LSI forms. The NC prioritized validation of survey information to the high-risk laboratories, those with inventory of poliovirus or poliovirus potentially infectious materials.

### Thoroughness of inventory process

Laboratories/biomedical facilities identified to keep poliovirus potentially infectious materials were given an inventory questionnaire for completion by the head of the laboratory. Thereafter, the ZC physically validated the completed inventory forms at the laboratories/biomedical facilities.

To further support the inclusion of such facilities in the national inventory list, the NTF members during their supervisory visitation to states physically validated the completed inventory of the laboratory. Finally, the NC internally and physically validated some of the laboratory information with completed Inventory and fully established their inclusion in the national Inventory list for the phase 1b activities.

A follow-up by means of reminder through phone calls, SMS and personally returning to the laboratories was done by the ZC, WHO staff and NC to enhance high response of 100% to the inventory questionnaire by all laboratories identified in the survey as holding poliovirus or poliovirus potentially infectious materials.

### Internal validation for destruction of poliovirus infectious and potentially infectious materials

An internal validation team consisting of a member of NTF, WHO, NC and Director, National Polio Reference Laboratory, visited and provided guidelines instructing the laboratories/biomedical facilities with inventory to destroy. The instructions were fully followed by heads of the laboratories.

The NC updates the national inventory list when identified laboratories are guided to destroy all poliovirus or poliovirus potentially infectious materials. In most instances the NC, with WHO staff or NTF member(s) and Director, WHO National Poliovirus Reference laboratory visits the identified laboratory, gives guidelines, and monitors the destruction process using the best standard practice. Such identified laboratory is then de-listed from the national inventory list.

Laboratories on the inventory that claimed to have destroyed all poliovirus potentially infectious materials were made to provide a validated signed copy of the inventory form to support their claim.

Laboratory report books indicating dates and methods of destruction of the poliovirus potentially infectious materials were provided to support such claim.

In the case of destruction of such poliovirus potentially infectious materials and, evidence of a functioning dirty autoclave must be seen by the NC and NTF members on supervisory visitation to establish appropriate implementation of best standard practice approach.

### Data analysis

Software (SPSS version 20) was used for data entry and analysis. The NC carried out data entry and also prepared weekly national data analysis, which was shared with WHO, NCC, National Primary Health Care Development Agency (NPHCDA), ZC and NTF.

## Results

In a bid to purge out poliovirus infectious materials, biomedical facilities identified as either high risk/missed or seen in phase 1a with inventory and new laboratories were surveyed in the containment exercise documented in this study.

Table 1 shows that a total of 560-biomedical facilities meeting the above mentioned criteria were surveyed in Nigeria in conformity with the GAP III. About 86% of the facilities surveyed were with laboratories while 14% of such facilities were without laboratories. New facilities found constituted 19% of the overall (560) biomedical facilities surveyed.

**Table 1:** Biomedical facilities surveyed across the zones in Nigeria

Zones	No. Of new facilities (%)	Type of facility		Total no. Of facilities (%)
		With laboratory (%)	Without laboratory (%)	
North central	4 (4)	103 (21)	1 (1)	104 (19)
North east	0 (0)	18 (4)	1 (1)	19 (3)
North west	33 (32)	73 (15)	17 (21)	90 (16)
South east	43 (41)	123 (26)	1 (1)	124 (22)
South-south	19 (18)	116 (24)	37 (46)	153 (27)
South west	5 (5)	47 (10)	23 (29)	70 (13)
Total	104 (19)	480 (86)	80 (14)	560

Total number of facilities surveyed was highest (27%) in the south-south zone and lowest (3%) in the northeast zone. The facilities in the southeast zone contributed the most to the total number of facilities with laboratories, followed by those in the south-south and north central zones while the north east facilities contributed the least. The facilities in the south-south zone contributed the most to the total number of facilities without laboratories, followed by those in the southwest and northwest zones while the northeast, southeast and north central facilities contributed the least (1 % each).

It should be noted that 99% of facilities surveyed in the north central, northeast and southeast zones were with laboratories.

A total of 12 laboratories with poliovirus potentially infectious materials were identified in this exercise (Table 2). While six of these laboratories were among those identified in the phase 1a exercise with poliovirus potentially infectious materials, two of them were new laboratories that were not listed in phase 1a. The remaining four laboratories with poliovirus potentially infectious materials were among those surveyed in the phase 1a exercise without any recorded inventory, but have acquired some since the phase 1a survey ended.

**Table 2:** Laboratories found with poliovirus infectious materials across the zones in Nigeria

Zone/States		Laboratories in Phase 1a with inventory still found with Poliovirus potentially infectious Material in Phase 1b	Laboratories not having Poliovirus Infectious Material in Phase 1a but now having Poliovirus potentiallyInfectious Material in Phase 1b	New Laboratories not identified in Phase 1a with Poliovirus Infectious Material	Total inventory found in labs
North Central	Plateau	0	0	1	1
North East	Borno	1	0	0	1
North west	0	0	0	0	0
South East	Enugu	1	0	0	1
South- South	Cross Rivers	0	0	1	1
	Rivers	1	1	0	2
	Edo	1	0	0	1
South West	Lagos	0	2	0	2
	Oyo	2	0	0	2
	Osun	0	1	0	1
Total		6	4	2	12

Facilities in the south west zone of the country contributed the most (five facilities) to the total number of facilities found with inventory followed by those in the south-south zone with three facilities, while those in the south east, north east and north central contributed the least (i.e. one facility each).

**Fig. 1:**
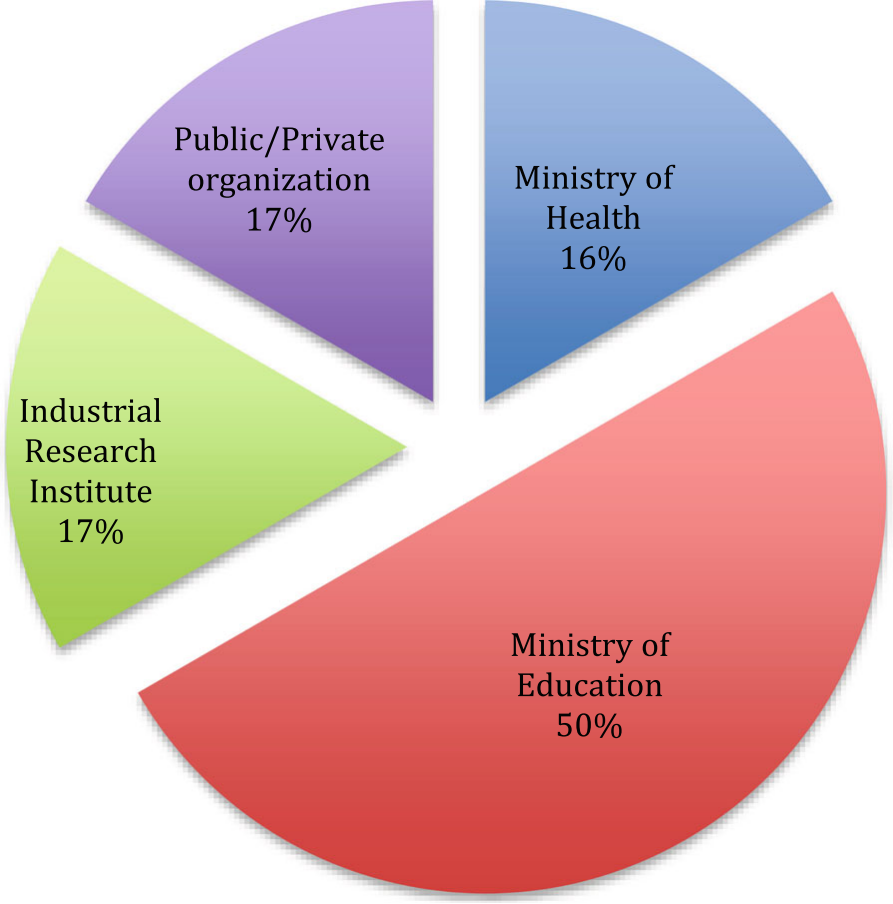
Biomedical facilities under Nigerian institutions where poliovirus infectious materials were found

However, no inventory was found in all the faculties surveyed in the northwest zone. Figure 1 shows 50% of the 12 laboratories found with poliovirus infectious materials were under the Nigerian Ministry of Education.

Classification/types of poliovirus infectious material found are detailed in Table 3. A total of 31,484 unwanted poliovirus infectious/potentially infectious materials were found during this survey.. 19,710 (62%) of these materials were stool sample closely followed by Non-polio enterovirus (NPEV) isolates, 9383 (30%) and Sabin vials isolates, 1851 (6%).

**Table 3:** Types of poliovirus infectious materials found in the laboratories across the zones in Nigeria

Zones	States	Type and Quantities of Poliovirus Infectious Materials Found							
		Stool	Cerebrospinal Fluid	Waste water	Rectal swap	Sewage	Non Polio Enterovirus isolates	Sabin vials isolates	Poliovirus isolates
North central	Plateau	1650	0	0	0	0	0	0	0
North east	Borno	7406	0	0	0	0	0	0	277
North west	0	0	0	0	0	0	0	0	0
South east	Enugu	0	20	0	0	0	0	0	0
South-south	Cross rivers	30	0	0	0	0	0	0	0
	Edo	0	26	0	0	0	0	0	0
	Rivers	50	0	5	0	0	0	0	0
South west	Lagos	50	0	0	50	0	0	0	0
	Osun	400	0	0	0	0	0	0	0
	Oyo	10,124	0	0	0	162	9383	1851	0
Total		19,710	46	5	50	162	9383	1851	277

Majority of the stool samples found came from Oyo, 10,124 (51%) and Borno sates, 7406 (38%) Oyo was the only state in the country to have stool sample, sewage waste, NPEV isolates and Sabin vials found in its laboratories. This was closely followed by Osun state in possession of both stool sample and rectal swap and Borno state with stool sample and poliovirus isolates in stock Other states in the zone had one of the above listed infectious materials except for the facilities in the northwest zone with zero poliovirus infectious materials found.

## Discussion

The implementation of part of GAP III’s phase 1b on the survey of national facilities for the containment/destruction of all unwanted poliovirus infectious materials/tOPV/mOPV2 helps minimise the risk of reintroducing laboratory associated polioviruses to the Nigerian population. This study documents survey activities and accesses Nigeria’s preparedness for poliovirus eradication in the process.

An in-depth revalidation exercise was carried out in 560 biomedical facilities, which were identified as either high or medium risk facilities in the Phase 1a exercise. This agrees with the approach used in a similar study done in the United States of America (USA) [11]. All the facilities surveyed in this particular exercise were completely (100%) validated and documented. The telephone call/text messages Follow up method used in this survey have also been used in other countries [12].

One of the major goals of this phase 1b activity is to make sure no laboratory holds any poliovirus potentially infectious materials in the country because, It has been clearly stated that the best way to prevent poliovirus from being reintroduced into a polio-free world is to completely destroy the virus and any potentially infectious materials related to it) [13]. As such, WHO recommends the complete destruction of such materials except where used for critical functions such as in vaccine production [14]. This however doesn’t apply in Nigeria.

Results show that 50% of the 12 laboratories fund with poliovirus infectious materials were under the ministry of Education including the polio reference laboratories located in both Maiduguri (Borno state) and Ibadan (Oyo state). This is in comparison to the phase 1a exercise where majority (58%) of the inventories found were in laboratories under the ministry of Health. Reasons adduced for this was that majority of the materials were being used for educational and research purposes. This is in consonance with findings from biomedical facilities survey done in the USA where academic laboratories possessed majority of potentially infectious materials [11]. This might however constitute a challenge to specimen management and containment in a developing nation like Nigeria with large volumes of student population characterized by consequent information loss during transition in academic laboratories) [15, 16]. Though the potentially infectious materials have since been destroyed, we recommend increased surveillance to ensure that unwanted poliovirus materials are reacquired in these facilities.

Results further indicates that four facilities previously not having any poliovirus infectious materials in the in the phase 1a were found with such materialsin the phase 1b exercise. The fact that some laboratories still acquiredpoliovirus infectious materials after destruction during the phase 1a containment indicates a certain level of weakness in the surveillance system and further buttresses the need for improved and continuous surveillance until the endgame strategic plan is totally achieved.

At completion of this phase 1b exercise, new facilities, which were hitherto not listed in the phase 1a activity, were now captured. In total, 104 new facilities were captured in this exercise, with majority (41%) of them found in the South East zone. It is interesting to note that this is similar to the findings obtained in the phase 1a exercise, where majority of the laboratories surveyed were in the South East. The reason for this might be due to early commencement of a degree programme in medical laboratory science in the zone and the entrepreneurial spirit of southerners.

In this exercise, a total of 31, 484 poliovirus potentially infectious materials were found albeit only 11,963 of such materials were immediately destroyed by autoclaving and incineration in compliance with the GAP III on containment.

The stool and sewage samples found in the Borno and Oyo states are scheduled for destruction within 3–4 months according to WHO protocol [17]. The Sabin isolates are also scheduled for destruction once the result of their genetic sequencing is received. Note that a combination of the samples found from these two states alone constitute 62% of the total poliovirus infectious materials found in this survey. Since there is no designated poliovirus essential facility (One with Bio-safety Level-3) in Nigeria, all poliovirus potentially infectious materials found in this survey are to be destroyed accordingly by autoclaving and incineration. We recommend that a supervisory visit be paid to the Oyo and Borno states laboratories to ensure compliance with destruction of the materials in their possession.

## Conclusion

This study has revealed the successful containment of all poliovirus infectious materials in the laboratories surveyed in preparation for poliovirus eradication. Surveillance gaps have also been indicated. We strongly recommend the improvement of the surveillance system in the country to maintain the gains from this exercise to avoid further reintroduction into any biomedical facilities. This reduces the chances of viral reintroduction to the population in general.

## Abbreviations

AFP: Acute Flaccid Paralysis
AMLSN: Association of Medical Laboratory Scientists in Nigeria
bOPV: bivalent Oral Polio Vaccine
cVDPV: circulating Vaccine-Derived Poliovirus
DSNO: Disease Surveillance and Notification Officer
GAP III: 3rd Global Action Plan
GCC: Global Certification Commission
HF: Health Facility
LSI: Laboratory Survey and Inventory
mOPV2: monovalent Oral Polio Vaccine type 2
NC: National Coordinator
NP: Non Polio
NPEV: Non Polio Enterovirus
NPHCDA: National Primary Health Care Development Agency
NTF: National Task Force
OPV2: Oral Polio Vaccine type 2
tOPV: trivalent Oral Polio Vaccine
USA: United States of America
WHO: World Health Organization
WPV: Wild Poliovirus
ZC: Zonal Consultant
